# Diphtheritic Arthritis

**Published:** 1885-02

**Authors:** 


*Diphtheritic Arthritis.*—During an epidemic of diphtheria, Pauli observed some cases of multiple articular pains as a complication of the disease. He explains this concomitance of the arthritis as the effect of the specific virus analogous to that of blenorrhagia.—*Paris Medical.*

